# PQBP1 regulates the cellular inflammation induced by avian reovirus and interacts with the viral p17 protein

**DOI:** 10.1016/j.virusres.2023.199119

**Published:** 2023-05-26

**Authors:** Chengcheng Zhang, Xinyi Liu, Qingqing Zhang, Jiahao Sun, Xiaorong Zhang, Yantao Wu

**Affiliations:** College of Veterinary Medicine, Yangzhou University, Jiangsu Co-Innovation Center for the Prevention and Control of Important Animal Infectious Disease and Zoonoses, Yangzhou, Jiangsu 225009, PR China

**Keywords:** Avian reovirus, P17 protein, PQBP1, Inflammation

## Abstract

Avian reovirus (ARV) can commonly infect a flock and cause immunosuppressive diseases in poultry. The nonstructural protein p17 is involved in viral replication, and significant progress has been made in showing its ability to regulate cellular signaling pathways. In our previous study, to further investigate the effect of ARV p17 protein on viral replication, the host protein polyglu-tamine binding protein 1 (PQBP1) was identified to interact with p17 by a yeast two-hybrid system. In the current study, the interaction between PQBP1 and p17 protein was further confirmed by laser confocal microscopy and coimmunoprecipitation assays. In addition, the N-terminal WWD of PQBP1 was found to mediate the process of binding to the p17 protein. Interestingly, we found that ARV infection significantly inhibited PQBP1 expression. While the quantity of ARV replication was largely influenced by PQBP1, PQBP1 overexpression decreased ARV replication. In contrast, upon PQBP1 knockdown, the quantity of ARV was notably increased. ARV infection and p17 protein expression were both proven to induce PQBP1 to mediate cellular inflammation. In the current study, we revealed through qRT‒PCR, ELISA and Western blotting methods that PQBP1 plays a positive role in ARV-induced inflammation. Furthermore, the mechanism of this process was shown to involve the NFκB-dependent transcription of inflammatory genes. In addition, PQBP1 was shown to regulate the phosphorylation of p65 protein. In conclusion, this research provides clues to elucidating the function of the p17 protein and the pathogenic mechanism of ARV, especially the cause of the inflammatory response. It also provides new ideas for the study of therapeutic targets of ARV.

## Highlights

• PQBP1 regulate the cellular inflammatory induced by avian reovirus.
• ARV influence the expression level of PQBP1 by the p17 protein.
• PQBP1 inhibits ARV replication.

## 1 Introduction

Avian reovirus (ARV) belongs to subgroup II of the genus *Orthoreovirus* and can cause viral arthritis, tenosynovitis, malabsorption syndrome, respiratory disease, enteropathy, impaired absorption syndrome, and osteoporosis in poultry (Zhang et al., 2021; Zhou et al., 2022b). ARV infection induces cellular innate immunity, leading to an inflammatory response against viral invasion (Xie et al., 2019). The ARV genome consists of 10 double-stranded RNA segments contained in a double-layered nucleocapsid, which can be classified into three groups: large (L1-L3), medium (M1-M3) and small (S1-S4). ARV genomes mainly encode 8 structural proteins (λA, λB, λC, μA, μB, σA, σB, σC) and 4 nonstructural proteins (μNS, p10, p17, σNS) (Benavente and Martinez-Costas, 2007; Martinez-Costas et al., 1997).

As a nucleoplasmic shuttling protein, p17, encoded by the S1 segment, is known to interfere with gene transcription and autophagosome activation (Li et al., 2015). Importantly, in our previous study, we showed that p17 is involved in the modulation of many cellular signaling pathways by interacting with host proteins (Zhang et al., 2022). Other studies revealed that p17 can activate p53, p21^cip1/waf1^, and the PI3K/AKT/mTOR and ERK signaling pathways (Huang et al., 2015; Liu et al., 2005). The p17 protein acts as a nucleoplasmic shuttle protein that can accumulate in the nucleus and plays a role in blocking signaling, modulating the host's immune response, and regulating interferon production.

Host cells recognize pathogens through pattern recognition receptors (PRRs) that sense ssRNA, dsRNA, and viral signature molecules (Hennessy and McKernan, 2021). ARV infection stimulates IFN production, transcription of proinflammatory factors, and downstream IFN-stimulated gene (ISG) synthesis by activating specific PRRs and interacting with interferon (IFN-β) promoter stimulator-1 (MAVS), which induces synthesis of antiviral proteins. In addition, transcription of IL-18 and IL-1β in the MDA5 signaling pathway causes acute inflammatory responses and exerts chemotactic effects on monocytes (Van Der Kraak et al., 2021). Additionally, ARV activates the NF-κB pathway through endocytosis, which contributes to the production of inflammatory factors and the delay of apoptosis (Xie et al., 2019). At the same time, it has also been proposed that duck reovirus (MDRV) can seriously damage the structure and function of the intestinal mucosa by regulating immune cells and immune-related factors (Li et al., 2020) and inhibiting the levels of IL-1β, IL-4, IL-5, and IL-18, resulting in local immunodeficiency (Wu et al., 2019).

In our previous study, the host protein polyglutamine binding protein 1 (PQBP1) was shown to interact with the p17 protein, and this interaction was further demonstrated by immunoprecipitation, cell colocalization and GST pulldown techniques (Zhang et al., 2022). PQBP1, which is localized in the nucleus (Nicolaescu et al., 2008), can be expressed in the central nervous system of embryonic and neonatal mice (Zhang et al., 2017). It acts as a novel regulator of translation elongation that can bind directly to eEF2 and affect its phosphorylation, control protein synthesis, influence mGluR signaling, and act as a participant in synaptic activity (Shen et al., 2021). In recent years, PQBP1 has attracted attention as a new immunoregulatory factor (Yoh et al., 2015). Most notably, a molecular mechanism similar to that described above was observed in microglia, the innate immune cells in the central nervous system (Jin et al., 2021). Tau protein, which is known to be involved in the pathogenesis of various neurodegenerative diseases, including AD and tauopathy, was found to activate microglia via the PQBP1-cGAS-STING pathway to promote brain inflammation, such as TNF, IL-6 and type 1 IFN (Jin et al., 2021). This finding demonstrates the potential of targeting PQBP1 as a new common therapy for neurodegenerative diseases. It plays an ameliorative role in the onset of neurodegeneration (Su and Shen, 2021). In cancer patients, overexpression of PQBP1 acts as a repressor of IFN-β promoter transcription produced by IFI16 and cGAS induction, resulting in blocked DNA signaling and reduced survival (Shannon et al., 2018). PQBP1 interacts with many proteins, including the U5 snRNP-specific 15 kDa protein (U5–15 kDa) and WBP11/NpwBP/SIPP1, and the KD or KO structure can significantly affect protein synthesis and is expected to be the next cellular therapeutic target for neurological diseases, viral infection and cancer (Mizuguchi et al., 2014).

In the current study, we further identified the WW domain that mediates the interaction with ARV p17 protein by a coimmunoprecipitation assay. The underlying molecular mechanisms of ARV-induced immunosuppression and inflammation correlated with the interaction between p17 and PQBP1 and the NF-κB signaling pathway. Furthermore, we revealed that PQBP1 can prominently inhibit ARV replication by overexpression and knockdown assays. These results can further expand our knowledge of ARV and increase the understanding of the functional role of the nonstructural protein p17.

## 2 Materials and methods

### 2.1 Reagents and plasmids

The pcDNA3.1 expression plasmid was bought from ThermoFisher (USA) and stored in our laboratory. Rabbit ARV p17 polyclonal antibody was obtained and stored in our lab. Restriction endonucleases *Bam* HI and *Eco* RI were purchased from NEB (USA). Trans1-T1 receptor cells, Taq high-fidelity DNA polymerase, pEASY-T3 cloning vector, DNA T4 ligase, and high-glucose DMEM were obtained from TransGen Biotech (Beijing, China). DL Marker and DNA gel recovery kits were purchased from TaKaRa (Dalian, China). The Animal Tissue RNA Extraction Kit and Plasmid Extraction Kit were purchased from Kang Wei Century (Beijing, China). The immunoprecipitation kit and AceQ Universal SYBR qPCR Master Mix were purchased from Novozymes Biotechnology Co. The mouse anti-Myc monoclonal antibody, rabbit anti-Flag monoclonal antibody and mouse anti-GAPDH monoclonal antibody were purchased from Beyotime Biotechnology (Beijing, China); Caspase-1 antibodies were purchased from Cell signaling technology; HRP-labeled sheep anti-mouse IgG and HRP-labeled sheep anti-rabbit IgG were purchased from Sigma; and goat anti-mouse IgG (*H* + *L*), FITC conjugate, and goat anti-rabbit IgG (*H* + *L*), PE conjugate, were purchased from TransGen (Beijing, China). FBS was purchased from LONSERA. 40,6-Diamino-2-phenylindole (DAPI) was purchased from Beyotime Company (Nanjing, China).

### 2.2 Virus and cell culture

DF-1, Vero and HEK293T cells were obtained from ATCC (USA) and grown in Dul-becco's modified Eagle's medium (DMEM) (Life Technologies Corp., Grand Island, NY, USA) supplemented with 10% (v/v) fetal bovine serum (FBS), 10 kU/ml penicillin and 1% 10 mg/ml streptomycin (50 IU/mL and 50 µg/mL, respectively, Sigma‒Aldrich, Burlington, MA, USA) and 250 μg/ml amphotericin B at 37 °C in a humidified atmosphere with 5% CO2. In this study, ARV strain GX/2010/1 (accession numbers KJ476699-KJ476708) was isolated by our lab and propagated in CEF cells, followed by three freeze‒thaw cycles. The supernatant was collected and stored at −80 °C. The virus titer was determined by plaque assay, and the cell culture infectious dose used was an MOI of 1.

### 2.3 Construction of recombinant plasmids

The CDS of ARV p17 was cloned from the genome of ARV GX/2010/1 with specific primers (shown in Table 1). The PQBP1 gene was originally cloned from cDNA extracted from DF-1 cells. All primers were synthesized by Sangon Company (Shanghai, China). Total RNA of the cells was extracted using the TRIzol kit and reversed transcribed to cDNA using SuperScriptase (Invitrogen, USA) following the manufacturer's instructions. The amplified PCR products were identified by 1% agarose gel electrophoresis. All the genes were verified by sequencing.

**Table 1:** Sequences of primers used in this article.

Primer name	Primer sequence(5′−3′)	Note
Myc-p17-F	GCGAATTCTATGCAATGGCTCCGCCATACG	Amplification of p17
Myc-p17-R	GCGGATCCTTACAGATCCTCTTCAGAGATGAGTTTCTGCTCCTCATGGATCGGCGTCAAA	gene with Myc label
qPQBP1-F	GGCATCCTCAAACATCTGG	Primers for detection of
qPQBP1-R Flag-PQBP1-F Flag-PQBP1-R Flag-WW-F Flag-WW-R Flag-PRD-F Flag-PRD-R Flag-CTD-F Flag-CTD-R IFN-β-F IFN-β-R cGAS -F cGAS -R Caspase-1-F Caspase-1-R IL-18-F IL-18-R ARV-F ARV-R β-actin-F β-actin-R si-PQBP1-S si-PQBP1-AS NC-siRNA-S NC-siRNA-AS	GCAGGAAGGGTCGAACAC CGGGATCCATGCCGCTGCCCGTT CGGAATTCTTACTTATCGTCGTCATCCTTGTAATCATCCTG CTGCTTGGTTCGGG CGGGATCCATGCCGCTGCCCGTT CGGAATTCTTACTTATCGTCGTCATCCTTGTAATCCTTGGCCGA TTTGGTAACCGCG CGGGATCCAAGCTCAGAAGCAGTAAT CGGAATTCTTACTTATCGTCGTCATCCTTGTAATCTT CTTTGCCCTCTTCCC CGGGATCCCGGCGCCACCATCG CGGAATTCTTACTTATCGTCGTCATCCTTGTAATCATC CTGCTGCTTGGTTCGGG CAAGTGTCTCCTCCAAAT AATTAGCCGGATGTGGTG ATGGAGTCTCGCTCTGTCG GAAGAGCAGAAAGCGATA TGAAGGACAAACCGAAGG ATCAGGAGGATTCATTTC ATAGCCAGCCTAGAGGTA CGTATCATTCACCCGCGATT TGTTCGCTGTACCATCACCT CTGTGCCCATCTATGAAGGCTA ATTTCTCTCTCGGCTGTGGTG GCCGAGGACUAUGACGAUGTT GAUCGUCAUAGUCCUCGGCTT UUCUCCGAACGUGUCACGUTT ACGUGACACGUUCGGAGAATT	PQBP1 expression Amplification of PQBP1 gene with Flag label Amplification of WWD gene with Flag label Amplification of PRD gene with Flag label Amplification of CTD gene with Flag label Primers for detection of IFN-β expression Primers for detection of cGAS expression Primers for detection of Caspase-1 expression Primers for detection of IL-18 expression Primers for detection of ARV replication Primers for detection of β-actin expression siRNA target of PQBP1 Negative control siRNA

The underline sequences represent the restriction enzyme cutting site.

### 2.4 Confocal microscopy

293T cells were cultured in 6-well plates at a density of ∼2 × 10^6^ cells/well and cotransfected with 3 μg of Flag-WWD and 3 μg of p17-Myc vector using TurboFect (Thermo Scientific, #R0531) according to the manufacturer's instructions. Forty-eight hours after transfection, the cells were washed with cold 1 × PBS, fixed with 4% paraformaldehyde for 10 min with 0.1% Triton X-100 at room temperature, and blocked with PBS containing 10% FBS. Immunohistochemical staining was performed by incubation with a mouse anti-Myc and rabbit anti-Flag antibody for 2 h at 37 °C, followed by incubation with goat anti-mouse IgG (*H* + *L*), FITC conjugate, and goat anti-rabbit IgG (*H* + *L*), PE Conjugate (TransGen), at 37 °C for 1 h. Nuclei were stained with DAPI at 37 °C for 20 min. Images were viewed by laser-scanning confocal microscopy (LSM510 META; Zeiss, Germany).

### 2.5 Coimmunoprecipitation and western blotting

Eukaryotic expression vectors for Flag-PQBP1 and Myc-p17 were cotransfected into DF-1 cells, and RIPA mixed with PMSF was used to collect cell protein samples after 48 h. The beads were pretreated with Myc antibody and incubated in a flip mixer. The antigen was incubated in combination with the treated bead-antibody complex at 4 °C overnight. After magnetic separation, the supernatant was discarded. The beads underwent repeated washes with wash buffer. Then, 1 × SDS‒PAGE buffer was added, the beads were subject-ed to a denaturating elution, and the supernatant was collected as the antigen sample, which was incubated with a rabbit anti-Flag primary antibody for detection by Western blotting. Cell lysates were prepared by using Pierce IP lysis buffer (25 mM Tris–HCl, pH 7.4, 150 mM NaCl, 1 mM EDTA, 1% NP-40 and 5% glycerol) containing the protease inhibitor phenylmethylsulfonyl fluoride for 30 min on ice. Then, the samples were separated by centrifugation at 12,000 rpm at 4 °C for 15 min, and the supernatants were further dena-tured by boiling with 10 × SDS‒PAGE loading buffer for 5 min. Twenty micrograms of pro-tein sample was used for the Western blotting assay. The protein bands of interest were developed using an enhanced chemiluminescence (ECL) kit. The concentrations of anti-bodies used were based on the manufacturer's instructions.

### 2.6 Transfection

PQBP1-specific siRNA oligonucleotides and scrambled siRNA (NC-siRNA) were synthesized by GenePharma (Shanghai, China). The sequences used are shown in Table 1. Vero cells grown to 70% confluency were transfected with siRNA or Flag-PQBP1 by using TransIntro EL Transfection Reagent (TransGen Biotech, Beijing, China) in a 6-well plate. At different times posttransfection, one group of cells was collected, and the mRNA levels of specific proteins were assessed by qRT‒PCR, while the remaining cells which after the transfection 48 h then were infected with ARV at an MOI of 1.

### 2.7 RNA extraction and quantitative real-time PCR (qRT-PCR) assay

Control-treated cells and cell samples were collected at different time points. Total cell RNA was extracted from Vero cells using TRIzol reagent (Invitrogen) and reverse tran-scribed to cDNA using SuperScriptase (Invitrogen) following the manufacturer's instruc-tions. The PCR conditions were 98 °C for 8 min, followed by 32 cycles of 94 °C for 25 s, 50 °C for 35 s, and 72 °C for 1 min, with a final step of 72 °C for 10 min. The transcript levels of ARV, PQPB1, IFN-β, IL-18 and caspase-1 were analyzed by qRT‒PCR. The spe-cific primers are described in Table 1, and the SYBR Green qPCR kit (Vazyme Biotech Co., China) was used. Data were analyzed using the comparative threshold cycle (CT) method. The housekeeping gene β-actin was used as a reference control.

### 2.8 ELISA experiments

The ELISA kits for detect the expression of IFN-β, IL-18 were purchased from Sero-tecAbD, and the experimental procedures were performed according to the manufactur-er's instructions. Briefly, the cells were scraped off, and the supernatant was repeatedly freeze‒thawed and added to the enzyme standard plate. Three replicate wells were set up for each sample, and blank wells were set up with standard wells. The enzyme standard reagent was added and incubated at 37 °C for 1 h. After washing, the color development solution was added, the cells were incubated in the dark for 15 min, the color devel-opment was terminated, and the cells were placed into the enzyme standard instrument for reading. After the enzyme plate was subjected to zymography for detection, the OD value observed at 450 nm was used as the vertical coordinate, and the infection time was used as the horizontal coordinate for graphical analysis.

### 2.9 Statistical analysis

The data in this study are expressed as the mean ± standard deviation (SD) and were assessed by Student's *t*-test or one-way ANOVA using GraphPad Prism 5 software. A *P* value of less than 0.05 was considered statistically significant. The relative expression ra-tios of the targeted proteins were analyzed by ImageJ software. All experiments were per-formed for three independent experiments.

## 3 Results

### 3.1 The ww domain (WWD) of PQBP1 interacts with arv p17

**Fig. 1:**
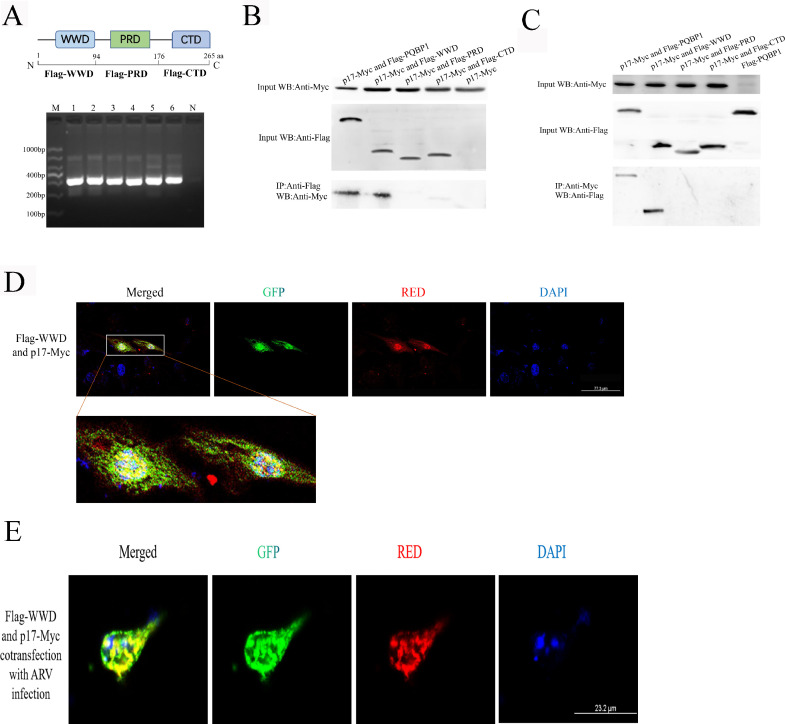
The N-terminus of PQBP1 interacts with the ARV p17 protein. (A) Schematic representation of the protein domains (shown as amino acids number) of PQBP1 and the PCR results of each domain. M, DL1000 marker; 1–2, Flag-WWD(313 bp); 3–4, Flag-PRD(298 bp); 5–6, Flag-CTD(313 bp); N, Negative control. (B) The N-terminal region of PQBP1 interacts with the p17 protein. 293T cells were transfected with different portions or the full-length form of PQBP1 with a Flag tag and p17-Myc plasmids for 48 h and harvested. Cell lysates were immunoprecipitated with an antibody against Flag followed by Western blotting analysis. (C) Reciprocal co-IP experiments showed that the anti-Myc antibody precipitated Flag-WWD. (D) Colocalization of WWD of PQBP1 and ARV p17 protein. 293T cells were cotransfected with Flag-WWD and p17-Myc for 24 h, then incubated with anti-Flag and anti-Myc primary antibodies and immunostained with PE- or FITC-labeled secondary antibodies. Finally, the cells were analyzed by confocal microscopy. (E) Colocalization of Flag-WWD and p17-Myc in the condition of ARV infection.

In a previous study, we identified that PQBP1 can interact with ARV p17 by immunoprecipitation and by a yeast two-hybrid system (Zhang et al., 2022). To dissect which region of PQBP1 is responsible for the interaction, we generated a series of expression fragments of PQBP1 according to the domain demarcation. PQBP1 proteins possess a WW domain (WWD), a polar amino acid-rich domain (PRD) with dinucleotide repeats and a C-terminal domain (CTD) with intrinsically disordered protein regions (Fig. 1A). PQBP1 has a WW domain (WWD) that is homologous to the SH3 domain in local protein structure and target sequence recognition (Kato et al., 2004) and a specific C-terminal domain (CTD) that is highly degenerated and classified as a low complexity domain/region (Wootton, 1994) or an intrinsically disordered protein (Schuler et al., 2020). We selected Flag-WWD (1–94 aa of PQBP1) (which included the WWD), Flag-PRD (94–176 aa of PQBP1) (which included the PRD), and Flag-CTD (193–265 aa of PQBP1) (which included the CTD). Based on the coimmunoprecipitation results, the WWD of PQBP1 accounted for the PQBP1 and p17 interaction (Fig. 1B).

To further confirm the interaction relationship between the WWD of PQBP1 and the p17 protein, we observed the cellular localization after cotransfection of the Flag-WWD and p17-Myc eukaryotic expression plasmids in 293T cells whether with or without ARV infecton, which demonstrated a yellow fluorescence that was distributed in a punctate form in the nucleus, as observed by confocal microscopy (Fig. 1D and 1E).

### 3.2 PQBP1 suppresses arv proliferation

**Fig. 2:**
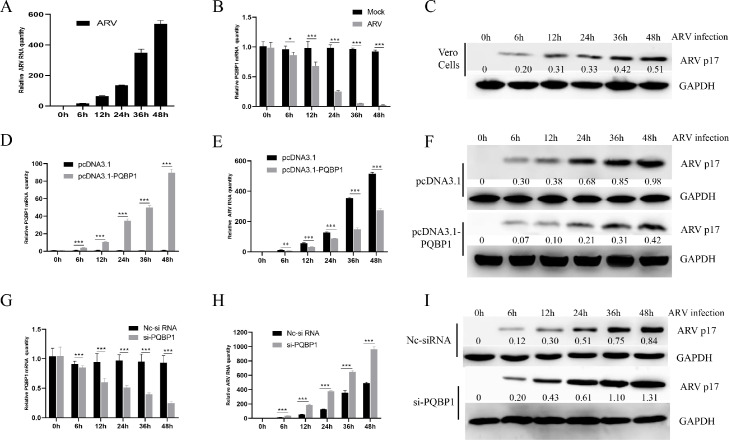
Cellular PQBP1 inhibits ARV proliferation. (A) Vero cells were infected with ARV at an MOI of 1 for different times, and the quantity of ARV replication was assayed by qRT‒PCR. (B) Intracellular PQBP1 expession after ARV infection was revealed by qRT‒PCR. (C) The ARV p17 protein levels in Vero cells were analyzed by Western blotting. The numbers represent the ratio of p17/GAPDH in different ARV infection time. (D) Intracellular PQBP1 expression after transfection with the pcDNA3.1-PQBP1 plasmid in Vero cells was revealed by qRT‒PCR. (E) After transfected with pcDNA3.1-PQBP1 48 h, the Vero cells infeciton ARV at indicated time. The quantity of ARV replication in Vero cells was revealed by qRT‒PCR. (F) The ARV p17 protein level in PQBP1-overexpressing Vero cells was analyzed by Western blotting. The numbers represent the ratio of p17/GAPDH in different ARV infection time. (G) Intracellular PQBP1 expression after transfection with si-PQBP1 or Nc-si RNA, then infection with ARV with MOI of 1 at different time in Vero cells was revealed by qRT‒PCR. (H) After transfected with si-PQBP1 48 h, the Vero cells infeciton ARV at indicated time. The quantity of ARV replication in Vero cells was revealed by qRT‒PCR. (I) The ARV p17 protein level in PQBP1-knockdown Vero cells was analyzed by Western blotting. The numbers represent the ratio of p17/GAPDH in different ARV infection time. Results are presented as the means ± SD of data from three independent experiments. (*P < 0.05, **P < 0.01; and ***P < 0.001).

**Fig. 3:**
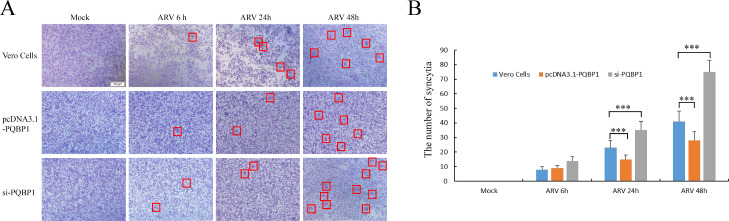
Cellular PQBP1 inhibits ARV proliferation in cell level. (A) The level of syncytium formation in Vero cells was detected by Giemsa-staining at different ARV infection times. Images were captured on an inverted microscope at 10 × objective. (B) The number of syncytia per microscopic field in Vero cells. Results are presented as the means ± SD of data from three independent experiments. ***, *P* < 0.001.

We further evaluated whether PQBP1 can affect ARV replication. Briefly, Vero cells were transfected with the overexpression plasmid pcDNA3.1-PQBP1, control plasmid (pcDNA3.1), a nontargeting siRNA (Nc-siRNA) or PQBP1-targeting siRNA (si-PQBP1). Then, the cells were infected with ARV at an MOI of 1 at different time points for analysis. First, we assessed ARV replication in Vero cells, as shown in Fig. 2A and C. Interestingly, ARV infection downregulated cellular PQBP1 expression (Fig. 2B). When PQBP1 was overexpressed (Fig. 2D) or downregulated (Fig. 2G) transfected with pcDNA3.1-PQBP1 and si-PQBP1, respectively, there was no difference in the amount of intracellular PQBP1 quantities between the pcDNA3.1 or NC-si RNA groups and the control infected group. However, the quantity of viral RNA (Fig. 2E) in infected ARV cells was significantly lower when PQBP1 was overexpressed and was increased when PQBP1 was knocked down compared with the control groups (Fig. 2E, F, H and I). The results of syncytium analysis in each group are shown in Fig. 3A, B. The data suggested that cellular PQBP1 inhibits ARV proliferation in cell level. These results showed that ARV can inhibit PQBP1 expression and the inhibitory effect of PQBP1 on virus proliferation.

### 3.3 PQBP1 can regulate the inflammatory factors induced by ARV infection

ARV has also been known to be the etiological agent of viral arthritis and tenosynovitis, which are related to the inflammatory response (Choi et al., 2022). In the current study, we determined the expression levels of inflammatory factors, such as IFN-β, IL-18 and caspase-1, in Vero cells infected with ARV or expressing with ARV p17 protein. As Fig. 3 shows, compared with mock infection or transfection with the empty plasmid pcDNA3.1, ARV infection or expression of p17 protein both significantly upregulated the expression of these inflammatory factors.

**Fig. 4:**
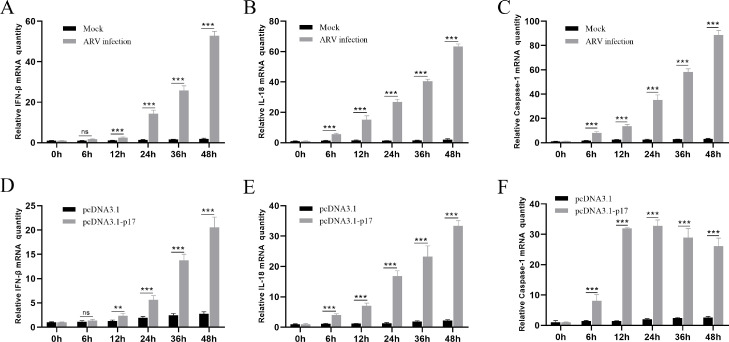
ARV infection and expression of p17 protein both induce cellular inflammation. Vero cells were infected with ARV at an MOI of 1 or expressing ARV p17 protein for different times, and the inflammatory factors were assayed by qRT-PCR. (A), (B) and (C) show the effect of ARV infection on the mRNA transcription levels of IFN-β, IL-18 and caspase-1, respectively. (D), (E) and (F) show the effect of ARV p17 protein expression on the mRNA transcription levels of IFN-β, IL-18 and caspase-1, respectively. Statistical analysis: unpaired t-test (*P < 0.05, **P < 0.01; and ***P < 0.001).

**Fig. 5:**
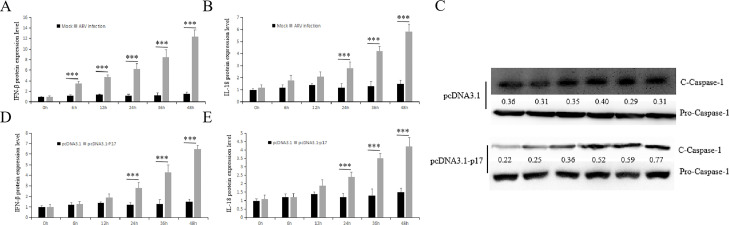
ARV infection and expression of p17 protein both induce cellular inflammation. Vero cells were infected with ARV at an MOI of 1 or expressing ARV p17 protein for different times, and the inflammatory factors were assayed by ELISA or Western blotting. (A) and (B) show the effect of ARV infection on the protein levels (ng/ul) of IFN-β and IL-18, respectively. (D), (E) and (C) show the effect of p17 protein expression on the protein levels (ng/ul) of IFN-β, IL-18 and Caspase-1, respectively. Results are presented as the means ± SD of data from three independent experiments. (***P < 0.001).

**Fig. 6:**
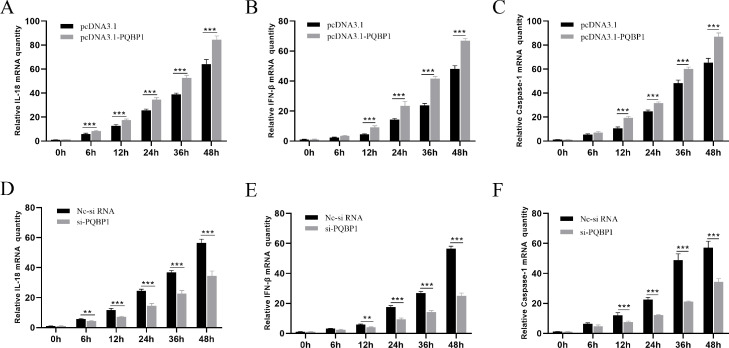
PQBP1 plays a positive role in ARV-induced inflammation. Vero cells with PQBP1 overexpression or knockdown were then infected with ARV at an MOI of 1 for different times, and the inflammatory factors were assayed by qRT-PCR. (A), (B) and (C) show the effect of PQBP1 overexpression on the mRNA transcription levels of IL-18, IFN-β and caspase-1, respectively. (D), (E) and (F) show the effect of PQBP1 knockdown on the mRNA transcription levels of IFN-β, IL-18 and caspase-1, respectively. Results are presented as the means ± SD of data from three independent experiments. (*P < 0.05, **P < 0.01; and ***P < 0.001).

PQBP1 is also an inflammation regulator that plays a role in the ARV-induced in-flammatory response. As Fig. 4, Fig. 5 shows, PQBP1 overexpression intensified the expression of inflammatory factors, while PQBP1 knockdown via siRNA led to the opposite results. To further confirm this result, ELISA and Western blotting were used to assess the protein expression level. As shown in Fig. 6, similar results showed that PQBP1 can regulate the inflammatory factors induced by ARV infection.

### 3.4 PQBP1 mediates ARV-induced nF-κB signaling pathway activation

**Fig. 7:**
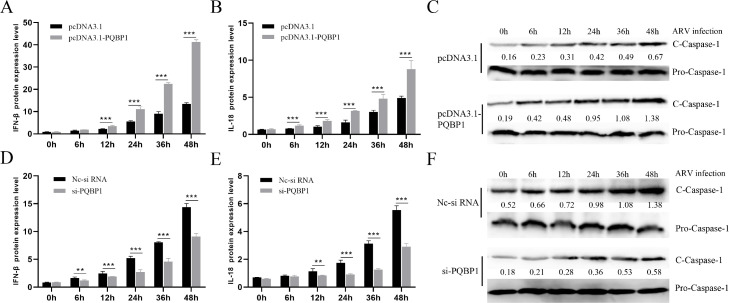
PQBP1 plays a positive role in ARV-induced inflammation. Vero cells with PQBP1 overexpression or knockdown were then infected with ARV at an MOI of 1 for different times, and the inflammatory factors were assayed by ELISA or Western blotting. (A), (B) and (C) show the effect of PQBP1 overexpression on the protein levels (ng/ul) of IFN-β, IL-18 and Caspase-1, respectively. (D), (E) and (F) show the effect of PQBP1 knockdown on the protein levels (ng/ul) of IFN-β, IL-18 and Caspase-1, respectively. Results are presented as the means ± SD of data from three independent experiments. (*P < 0.05, **P < 0.01; and ***P < 0.001).

**Fig. 8:**
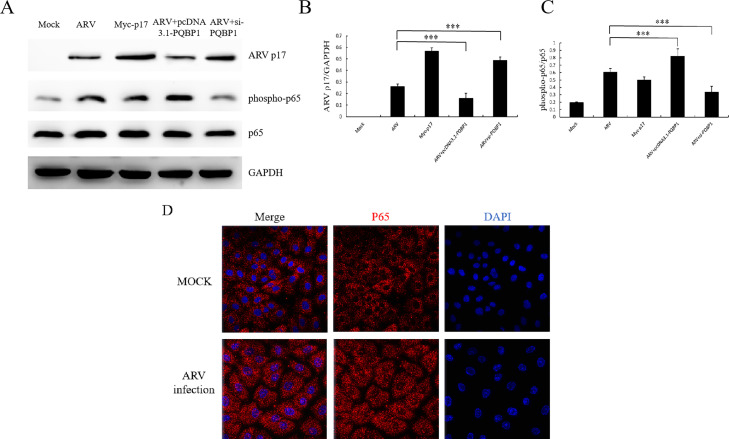
PQBP1 mediates ARV-induced NF-κB activation in Vero cells. (A) Western blots reveal activation of NFκB (phospho-p65) after ARV infection, p17 protein expression or overexpression or PQBP1 knockdown in Vero cells. (B) Relative p17 protein levels are presented. (C) Relative phospho-p65 protein levels are presented. This experiment was repeated independently three times with similar results. (D) Immunofluorescence images of Vero cells stained for p65 after ARV infection 24 h or mock infection. Scale bars: 49.1 µm. Results are presented as the means ± SD of data from three independent experiments. (***P < 0.001).

PQBP1 functions in innate immune cells as an intracellular receptor that recognizes pathogens and neurodegenerative proteins (Tanaka and Okazawa, 2022). In the current study, we showed that PQBP1 can regulate inflammatory factors, such as IL-18 and IFN-β, induced by ARV infection. Whether the mechanism is related to NF-κB is not known. Therefore, we determined the level of phospho-p65 in PQBP1-overexpressing or PQBP1-knockdown Vero cells infected with ARV. As shown in Fig. 7, we reconfirmed that PQBP1 inhibits ARV replication. PQBP1 plays a positive role in the ARV-induced NF-κB signaling pathway. We found that p65 phosphorylation can be induced by p17, overexpression of PQBP1 causes an increase in the active form of NF-κB, and the deletion of PQBP1 greatly inhibits the phosphorylation level of p65 (Fig. 8A–C). In order to study the effect of ARV on p65 nuclear translocation directly, the cellular localization of p65 in Vero cells were observed by immunofluorescence microscopy. As the Fig. 8D shown, endogenous p65 protein was distributed in the cytoplasm. Upon ARV infection, the endogenous p65 protein was distributed in both the cytoplasm and nucleus.

## 4 Discussion

ARV infection causes atrophy of the chicken bursa and thymus gland, connective tissue hyperplasia, heterophilic cell, and lymphocyte infiltration. Different types of inflammatory factors are expressed in different stages of ARV infection of cells and tissues (Clarke et al., 2005; Huang et al., 2022). Apoptosis caused by autophagy in immune organs is a common outcome of ARV infection. After inducing autophagy in experiments with the autophagy stimulator rapamycin, it was found that the strengthening of autophagy in cells promoted the proliferation of ARV (Zhou et al., 2022a).

The p17 protein contributes significantly to initiating autophagy through signaling pathways such as p53-PTEN-mTORCL, AMPK, and PKR/eIF2 to promote viral replication (Huang et al., 2015). It is particularly important to study and analyze the interaction host protein of the p17 protein and to understand the pathogenesis of ARV. Using a yeast two hybrid system, we identified 19 host proteins that can interact with the p17 protein. Among these proteins, PQBP1 functions in innate immune cells as an intracellular receptor that recognizes pathogens and neurodegenerative proteins (Tanaka and Okazawa, 2022). PQBP1 is highly conserved in evolution, containing a folded WW domain that mediates specific protein interactions through proline-rich or proline-containing short motifs(Sudol et al., 2012). It regulates RNA splicing, transcription, and DNA damage repair under physiological conditions(Mizuguchi et al., 2014); it also acts as a sensor protein for external molecules in macrophages and homologous cells of the innate immune system under certain pathological conditions (Yoh et al., 2015). The PQBP1 gene is widely expressed, and its various domains play different functions in the cytoplasm and nucleus. In the cell colocalization experiment (Fig. 1), we further confirmed that the WWD of PQBP1 mediates the interaction with the ARV p17 protein.

We found that the expression level of PQBP1 significantly decreased in a time-dependent manner after ARV infection (Fig. 2). Interestingly, the proliferation of ARV was upregulated by PQBP1 overexpression but downregulated by PQBP1 knockdown in Vero cells. We conclude that PQBP1 has a negative regulatory effect on the proliferation of ARV. Therefore, when ARV infects host cells, to ensure its replication, ARV inhibits the expression of PQBP1. PQBP1 may be involved in an innate immune response after ARV infection. As shown in Fig. 3, the expression of IFN-β, IL-18 and caspase-1 was upregulated by ARV infection and p17 protein expression. PQBP1 acts as a DNA sensor that binds to host DNA ligands to sense cellular stress or damage. Mitochondrial DNA release due to cellular stress or viral infection leads to the production of interferons and other antiviral responses that rely on cytosol-based DNA sensors (Drummond et al., 2003). A previous experiment on HIV infection showed that PQBP1 can act as a sensor receptor activating the cGAS receptor in complexes, inducing transcription of inflammatory factors and cytokine production, and directly involving cells with exogenous or endogenous antigens (He et al., 2022). Caspases (cysteine-requiring aspartate protease) are a family of proteases that play an important role in the process of apoptosis. Caspase-1, also known as interkeukin 1b converting enzyme (ICE), sometimes written as caspase 1, is the only caspase in the caspase family that can cleave IL-1b precursor protein or IL-18 precursor to produce corresponding mature cytokines. We found that the expression of the inflammatory factors IFN-β, caspase-1, and IL-18 was significantly different in the PQBP1 overexpression group compared with the control group. In addition, as shown in Figs. 6 and 7, upregulation of PQBP1 expression was critical for nuclear transposition that senses NF-κB, as well as the expression of inflammatory genes(Jin et al., 2021). The Western blot results showed that upregulation of PQBP1 caused an increase in the active form of NF-κB (pp65); however, this promoting effect was suppressed by silencing PQBP1. However, the mechanism of action involving PQBP1 and the p17 protein is not well understood. Further experiments are needed to study whether the p17 protein plays a role in this process. However, it is undeniable that PQBP1 is a cell receptor in the NF-κB pathway that induces inflammation.

In conclusion, this study provides clues for elucidating the function of the p17 protein and the pathogenic mechanism of ARV, especially the cause of the inflammatory response. It also provides important implications for our understanding of the pathogenic mechanism of ARV and the occurrence of inflammation.

## CRediT authorship contribution statement

**Chengcheng Zhang:** Conceptualization, Investigation, Writing – original draft, Writing – review & editing. **Xinyi Liu:** Conceptualization, Investigation, Writing – original draft, Writing – review & editing. **Qingqing Zhang:** Investigation, Writing – original draft, Writing – review & editing. **Jiahao Sun:** Formal analysis, Writing – original draft, Writing – review & editing. **Xiaorong Zhang:** Formal analysis, Writing – original draft, Writing – review & editing. **Yantao Wu:** Writing – original draft, Writing – review & editing.

## Declaration of Competing Interest

The authors declare that they have no conflict of interest!

## Data Availability

Data will be made available on request.
